# Genome Sequence of a Virulent Genotype III Newcastle Disease Virus Isolated from Laying Ducks in China

**DOI:** 10.1128/genomeA.01436-16

**Published:** 2016-12-29

**Authors:** Guoyuan Wen, Min Wang, Honglin Wang, Lintao Li, Qingping Luo, Tengfei Zhang, Guofu Cheng, Huabin Shao

**Affiliations:** a Institute of Animal Husbandry and Veterinary Sciences, Hubei Academy of Agricultural Sciences, Wuhan, China; b College of Veterinary Medicine, Huazhong Agricultural University, Wuhan, China; c Hubei Key Laboratory of Animal Embryo and Molecular Breeding, Wuhan, China

## Abstract

Here, we report the complete genome sequence of a virulent Newcastle disease virus (NDV) strain HN1007, isolated from diseased duck flocks in Henan, China, in 2010. The isolate has a genome length of 15,186 nucleotides, and was classified as a member of genotype III of class II.

## GENOME ANNOUNCEMENT

Newcastle disease (ND) is a highly contagious and often fatal disease of birds, caused by the virulent strains of Newcastle disease virus (NDV), also well known as avian paramyxovirus type 1. NDV owns a single-stranded, nonsegmented RNA genome of negative polarity, and belongs to the genus *Avulavirus* within the family *Paramyxoviridae* (1). Although it represents a single serotype, NDV has at least 19 different genotypes divided into two distinct classes based on genetic analyses. Waterfowl were considered to be the natural reservoirs of NDV, and generally showed no clinical signs of ND when infected with even highly virulent NDV (2, 3). A number of NDVs have been isolated from ducks, including genotypes I, II, VII, or IX, and XVII (4–6). However, ND outbreaks in duck flocks aroused much concern during the past decade in China, and most of these isolates belong to genotype VII (7, 8).

A laying duck farm suffered from a disease outbreak in Henan, China, in 2010. The morbidity and mortality of the affected flocks was about 30% and 10%, respectively, and the egg production greatly decreased by 50%. The diseased ducks showed clinical signs with difficultly breathing, diarrhea, torticollis, and paralysis. NDV strain HN1007 was isolated from a trachea-lung suspension by inoculating nine-day-old specific-pathogen-free chicken embryos.

To evaluate the virulence of NDV strain HN1007, pathogenicity index tests were carried out. The results demonstrated that the isolate was classified as velogenic NDV with a mean death time of 53 h and an intracerebral pathogenicity index of 1.94, respectively. The isolate has the virulent F protein cleavage site sequence (112R-R-Q-R-R-F117), which is consistent with the result of the in-vivo pathogenicity test.

For the whole-genome sequencing, eight pairs of specific primers were designed and utilized to amplify the genomic fragments covering the entire genome of the HN1007 virus. The eight overlapping reverse transcription-PCR (RT-PCR) products were purified and sequenced by Sangon Biotech (Shanghai, China). The complete genome of the HN1007 virus was aligned and assembled by SeqMan, and found to be 15,186 nucleotides in length. After phylogenetic analysis, the isolate was categorized into genotype III of the class II branch, the group of viruses caused the first pandemic outbreak of NDV in the 1930s. Compared to the genome sequences of other published NDV strains, there was 72.35% to 99.84% homology at the nucleotide level. To be specific, the isolate had the highest sequence homology (99.84%) to NDV strain Mukteswar (GenBank accession no. JF950509). The nucleotide sequence identities of the six structural genes (nucleoprotein, phosphoprotein, matrix, fusion, hemagglutinin-neuraminidase, and large polymerase protein) between the isolate and the Mukteswar strain were 99.89%, 99.86%, 99.68%, 99.78%, 99.80%, and 99.87%, respectively.

We reported the identification and complete genome sequence of the virulent genotype III NDV strain isolated from ducks in China. It will help us to better understand the epidemiology and evolution of NDV in waterfowl.

### Accession number(s).

The complete genome sequence of the NDV strain HN1007 has been deposited to GenBank under the accession no. KX761866.
